# Peripheral Refraction and Visual Function of Novel Perifocal Ophthalmic Lens for the Control of Myopia Progression

**DOI:** 10.3390/jcm12041435

**Published:** 2023-02-10

**Authors:** Sara Silva-Leite, Ana Amorim-de-Sousa, António Queirós, José Manuel González-Méijome, Paulo Fernandes

**Affiliations:** 1 Clinical & Experimental Optometry Research Lab (CEORLab), School of Sciences, University of Minho, 4710-057 Braga, Portugal; 2 Physics Center of Minho and Porto Universities (CF-UM-UP), 4710-057 Braga, Portugal

**Keywords:** myopia control, perifocal ophthalmic lens, peripheral refraction, visual contrast sensitivity, light disturbance

## Abstract

This study aimed to evaluate the peripheral defocus induced with a novel perifocal ophthalmic lens for myopia progression control and the potential impact on visual function. This experimental, non-dispensing crossover study evaluated 17 myopic young adults. The peripheral refraction was measured using an open-field autorefractor, at 2.50 m from the target point, in two eccentric points, 25° temporal, 25° nasal, and central vision. Visual contrast sensitivity (VCS) was measured at 3.00 m with a Vistech system VCTS 6500 in low light conditions. Light disturbance (LD) was assessed with a light distortion analyzer 2.00 m away from the device. Peripheral refraction, VCS, and LD were assessed with a monofocal lens and perifocal lens (with an add power of +2.50 D on the temporal side of the lens, and +2.00 D on the nasal side). The results showed that the perifocal lenses induced an average myopic defocus of −0.42 ± 0.38 D (*p*-value < 0.001) in the nasal retina, at 25° The changes induced by the lower add power in the nasal part of the lens did not induce statistically significant changes in the refraction of the temporal retina. The VCS and LD showed no significant differences between the monofocal and perifocal lenses.

## 1. Introduction

Myopia is a public health concern, and is estimated that by 2050, 50% of the world population will be myopic, with 10% of these being high myopic with −5.00 D or higher [1]. High myopic patients are at greater risk of developing ocular diseases such as glaucoma [2], cataracts [3,4], retinal detachment [5], and myopic maculopathy [6] or myopic macular degeneration [7], which can lead to visual impairment [8].

Currently, several interventions are available to manage myopia progression, all of them with different success rates. Optical interventions include different lenses with specific designs for the control of myopia progression. In a two-year randomized clinical trial, the defocus incorporated multiple segments (DIMS) lens showed a reduction in myopia progression of 0.55 D in the spherical equivalent error (SER) and 0.32 mm in the axial length (AL) compared with a control group [9]. With the DIMS lens, visual contrast sensitivity (VCS), showed no decrease in VCS compared with a single-vision lens, in both photopic and mesopic conditions [10].

Another optical lens design, the highly aspherical lenslets (HAL) and slightly aspherical lenslets (SAL) showed a reduction of 0.80 D in SER and 0.25 mm in AL, and 0.42 D in SER and 0.18 mm in AL, respectively, compared with the control group in a two-year randomized clinical trial [11]. A study that evaluated visual contrast sensitivity with these three lenses showed a smaller impact on VCS with HAL and SAL lenses than with DIMS lenses [12].

Recently, perifocal lenses were introduced. These new ophthalmic lenses have a central correction zone surrounded by an increasingly positive treatment zone on the nasal and temporal sides to reduce peripheral hyperopia. The effectiveness of this lens was reported in a Russian-based study where the test group was 0.79 D less myopic than a control group after four years of follow-up. They found that the peripheral refraction was more myopic at the 15° nasal as well as 15° and 30° temporal zone [13]. Nevertheless, the actual effect of this lens on peripheral refraction and the potential impact on visual quality is not known. To the best knowledge of the authors, the present study is the first to evaluate this new spectacle design intended for myopia management.

With that in mind, it was hypothesized that the optical design of the perifocal lens might have an impact on visual quality, due to the myopic defocus induced along the horizontal nasal and temporal areas of the lens. The purpose of this study was to analyze the visual function of this novel method to control myopia progression by comparing the peripheral refraction, visual contrast sensitivity, and light disturbance (LD) between monofocal lenses and perifocal lenses.

## 2. Materials and Methods

### 2.1. Study Design

This was a non-dispensing, non-randomized, and non-blinded experimental crossover study where young adult myopes wore perifocal lenses for the analysis of peripheral refraction, VCS, and LD. These measurements were made at 2 different moments. Firstly, the control measurements with monofocal lenses from the trial lens set, followed by the same examinations with the perifocal lenses.

This study was conducted at the Clinical and Experimental Optometry Research Laboratory (CEORLab) at School of Sciences from University of Minho (Braga, Portugal). Written informed consent was obtained from all the participants before the enrolment, and the study was performed according to the principles of the Declaration of Helsinki. This study was approved by the ethics committee of the University of Minho (CEICVS) which analysed the ethical aspects of this study to help protect the rights and well-being of the participants (CEICVS number 113/2022).

### 2.2. Eligibility Criteria

All the participants included in this study complied with the selected eligibility criteria. The inclusion criteria were young healthy subjects between 18 and 35 years, with a negative spherical refractive error inferior to 6.00 D, and astigmatism below 3.00 D (excluded). Participants with systemic diseases affecting ocular health, with eye diseases (e.g., keratoconus), and history of previous eye surgeries or anisometropia above 1.50 D were excluded.

### 2.3. Perifocal Lens

**Figure 1:**
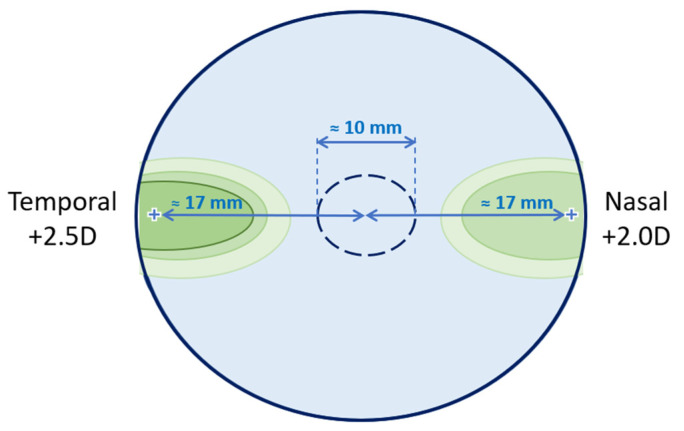
Schematic figure of the lens design according to the manufacturer. This represents the lens for the right eye, at 38 mm in diameter. The central monofocal correction circle is 10 mm in diameter.

The perifocal lenses (test condition) used have a uniformly powered oval area (≈10 mm wide) to correct central distance refraction, with a treatment area on each side of the central area in the horizontal plane intended to change the peripheral refraction. There is a positive increase from the center to the periphery to a maximum value of +2.50 D at 17 mm on the temporal side and +2.00 D at 17 mm on the nasal side of the lens, as shown in Figure 1. The temporal side of the lens has a higher treatment power than the nasal side intended to compensate for the asymmetries of the retinal contour between the nasal and temporal sides [14].

In the present study, we used 4 perifocal lenses with different central power: 0.00 D, −1.50 D, −3.50 D, and −5.50 D. After the subjective refraction, the lens with the closest correction to the refractive error of the participant was selected between the 4 lenses mentioned. Perifocal test lenses were glazed into circular shapes to adapt to the trial frame (total diameter of 38 mm), covering the central part and the temporal and nasal treatment areas of the lens. For each participant, 3 lenses were placed in the trial frame, in both control and test conditions. The first, closer to the eye, was the test (perifocal) lens previously selected, the second one, in the middle, was the residual spherical correction, and the third was the cylindrical lens to allow the total correction of the refractive error of the participant. When the refractive error of the participant was only spherical, a third plano lens was placed in the trial frame to maintain the same conditions in all participants.

### 2.4. Clinical Assessments

The axial length was measured with an IOL Master (Carl Zeiss Meditec, Inc., Dublin, CA, USA). After that, the refractive error of the participant was determined through an autorefractor followed by retinoscopy and subjective sphero-cylindrical refraction to obtain the maximum positive, which also ensured the best visual acuity. Best corrected visual acuity (BCVA) was measured with the ETDRS high-contrast chart (105 cd/m^2^) under photopic conditions (150 lux) measured with the Luminance Meter LS110 and Illuminance Meter T10 (Konica Minolta Sensing, Inc., Osaka, Japan), respectively. The interpupillary distance was obtained with the Pupilometer HX-400 (Chongqing Yeasn Science and Technology Co., Chongqing, China). The measurements for the control condition were obtained with the participant fully corrected in the trial frame. These measurements included peripheral refraction, contrast sensitivity, and light disturbance analysis. Thereafter, these measurements were repeated with the perifocal lenses.

### 2.5. Peripheral Refraction

**Figure 2:**
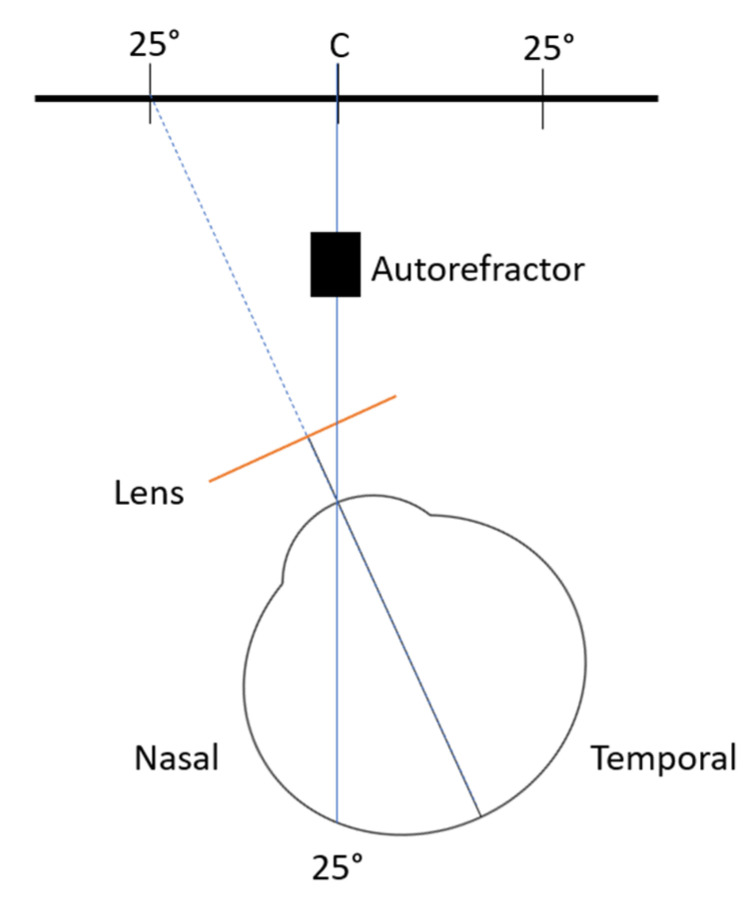
Schematic illustration of the measurement of the peripheral refraction at 25° temporal retina of the right eye. Not to scale.

Peripheral refractive error was measured with an open-field autorefractor (WAM-5500, Grand Seiko Co, Lda, Hiroshima, Japan) at 2.50 m from the target point. The target point was a star in the wall that allows us to measure the peripheral refractive error at approximately 25° nasal (25N) and 25° temporal (25T) of retinal eccentricity. The autorefractor obtains 5 measurements at each point and calculates the mean value of these measures. Figure 2 illustrates how the measures were obtained when the participants were rotating the head to assess the refraction at the 25° temporal retinal side of the right eye, measured through the nasal side of the lens. The measures were obtained monocularly, with the contralateral eye occluded and ambient light conditions (19 cd/m^2^ and 160 lux).

Instead of rotating the eye to fixate on different targets, as is usually the case in the naked eye, eyes wearing contact lenses, or in orthokeratology studies [15,16], in this study the measurements were undertaken by rotating the head to keep the eyes aligned with the control or test lenses in all situations. To control the alignment, the participants used a hair bow with a fixed laser, to ensure that the measurements were taken at the required point of eccentricity while turning their heads.

To analyze the refraction data, the sphere cylinder and axis values obtained from the autorefractor were converted in vectorial components into spherical equivalents (*M*), astigmatic components in the horizontal meridian (*J*0), and oblique astigmatic components (*J*45), according to Thibos et al. (1997) [17]. The M component is calculated by adding half of the cylinder to the sphere (Equation (1)). The second value, *J*0, expresses the differences between the horizontal and vertical meridian in terms of diopters, and this value is negative to against-the-rule astigmatisms and positive to with-the-rule astigmatisms (Equation (2)). The *J*45 describes the value from oblique astigmatisms, being negative to astigmatisms whereby the negative axis is at 135° or positive to astigmatisms whereby the positive axis is at 45° (Equation (3)).

(1) $$M=sphere+\frac{cylinder}{2}$$

(2) $$J0=-\frac{cylinder}{2}\times \cos\left(2\times axis\right)$$

(3) $$J45=-\frac{cylinder}{2}\times \sin\left(2\times axis\right)$$

The peripheral refraction is presented in relative values, in which the central value was subtracted for each peripheral value for *M*, *J*0, and *J*45. After this, the tangential and sagittal focals (*F_T_* and *F_S_*, respectively) were calculated using Equations (4) and (5):

(4) $$F_{T}=M+J0$$

(5) $$F_{S}=M-J0$$

### 2.6. Contrast Sensitivity

The contrast sensitivity was measured using a Vistech system VCTS 6500 (Vistech Consultants, Dayton, OH, USA), in low light conditions. The VCTS is composed of 5 lines representing different spatial frequencies (1.5; 3; 6; 12 and 18 cycles/degree—cpd). Each line is composed of 9 circles, with different bar orientations (vertical, right-oriented, or left-oriented), whose contrast progressively decrease up to number 9. To simulate the vision under challenging conditions, the luminance of the test was 15 cd/m^2^ and the illuminance of the room was 29 lux.

The participants were 3.00 m away from the chart, with an occluded eye, and were asked to state the orientation of the bars in each line until they were not able to recognize it. The number of the last circle visible in each line was registered to determine the contrast sensitivity according to the 5 spatial frequencies assessed. For the purpose of analysis, contrast sensitivity was converted to log contrast sensitivity.

### 2.7. Light Disturbance

**Figure 3:**
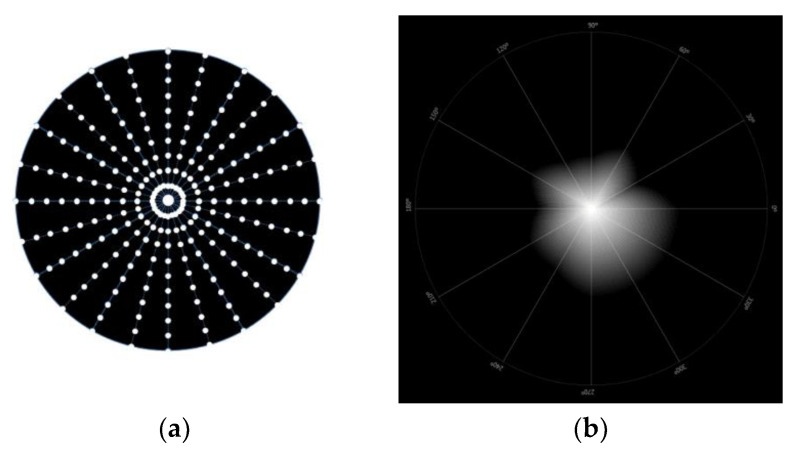
Representative images of LDA device. (**a**) Representative figure of the LDA system; (**b**) simulated image of the light disturbance of one measure, presented by the LDA device.

Light disturbance was obtained using a light distortion analyzer (LDA, Binarytarget Lda., Braga, Portugal). This device has a circular electronic black board with a central light spot with high-intensity light [18]. This central light is an LED that is surrounded by 240 smaller and less intense LEDs distributed over 24 semi-meridians with an angular separation of 15°, as demonstrated in Figure 3 below. The central LED is responsible for creating the glare condition while the peripheral LEDs are used as limit discriminators of that condition at separate locations in the visual field. These physical LEDs allow the measurement of some parameters of light disturbance under more realistic conditions [19].

The central LED is always on, and the peripheral LEDs light up sequentially throughout the meridians while the participant presses the computer mouse button whenever they are able to identify the light of one of the peripheral LEDs. When that happens, the system changes for the evaluation of the next semi-meridian, and the process is repeated until all semi-meridians are evaluated. The participant is placed 2.00 m away from the electronic board with its visual system aligned with the central LED. The exam was performed under low-lightning conditions, and the measurements were obtained monocularly, with the best distance visual correction.

In this study, the in-out 30° strategy was used, where the peripheral LEDs turn on sequentially from the center to the periphery in a random order with an angular separation between the semi-meridians of 30°. Three evaluations are performed in each meridian with approximately 1 minute per exam. In cases where the standard deviation (SD) of the 3 measurements are 20% above the mean, the system repeats the measurements until it obtains an SD below 20% of the mean value for each meridian [19].

The software provides 2 metrics that allow the quantify the size of the light disturbance, the light disturbance index (LDI), and the best-fit circle (BFC) radius (BFC_Rad_), and 2 metrics that evaluate the irregularity of the distortion, the BFC irregularity (BFC_Irreg_) and the standard deviation of the BFC irregularity (BFC_IrregSD_). LDI is estimated by the ratio of the area missed by the participant and the total area explored, presented in percentage (%). Higher values of LDI are interpreted as the lower ability to discriminate small stimuli surrounding the central source of light. BFC_Rad_ is the radius of a circle that best fits the shape of the disturbance area and is expressed in millimeters (mm). The sum of the deviations between the actual disturbance area and the BFC outer perimeter along with all the semi-meridians assessed is the BFC_Irreg_, presented in mm. Finally, the BFC_IrregSD_ is the sum of the differences squared and divided by the number of semi-meridians evaluated, expressed in mm. Higher values of BFC_IrregSD_ mean a more irregular disturbance [19].

### 2.8. Statistical Analysis

The statistical analysis was performed with SPSS statistic software version 29.0 (SPSS Inc., Chicago, IL, USA). The descriptive data obtained are presented in the form of mean ± standard deviation. The normality of variables was evaluated using the Shapiro–Wilk test since the sample was lower than 30 subjects. When both variables (control and test) of the peripheral refraction, contrast sensitivity, and light disturbance followed a normal distribution, a paired samples *t*-test was used. A Wilcoxon test was used when at least one of the variables did not follow a normal distribution. A *p*-value lower than 0.05 was considered statistically significant, marked in bold in the tables presented in the results section.

## 3. Results

### 3.1. Sample Characterization

Seventeen participants were included, with a mean age of 24.0 ± 3.5 and mean spherical refraction of −2.80 ± 1.75; 14 (82.4%) were female and 3 (17.6%) were male. A primary statistical analysis showed no differences between the right and left eyes, therefore only the right eyes were included for statistical analysis. Table 1 shows the characteristics of the subjects in terms of age, gender, refractive error, best-corrected visual acuity (BCVA), and axial length.

### 3.2. Peripheral Refraction

**Figure 4:**
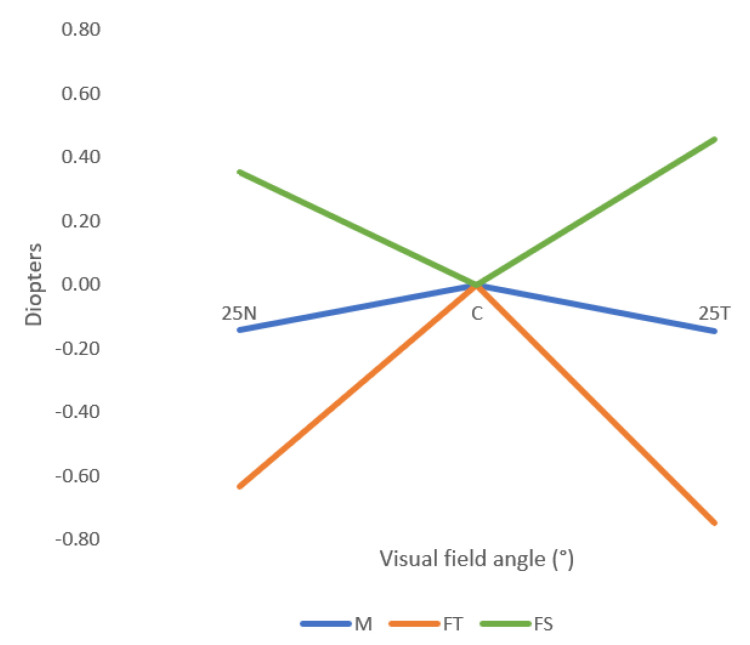
Mean values of the relative spherical equivalent (M), the sagittal focal (FS) and the tangential focal (FT), without any correction (naked eye condition). 25N: 25° eccentricity at nasal retina, 25T: 25° eccentricity at temporal retina. Standard deviation is not shown for clarity and can be consulted in Table 2.

Figure 4 shows the relative peripheral refraction in terms of spherical equivalent (M), tangential focal (F_T_) and sagittal focal (F_S_) of the right eye of the participants, without any correction of the refractive error (naked eye).

Table 2 describes the relative mean values of the M, FT, and FS for the right eye. The M values measured at 25N were significantly different (*p*-value < 0.001, paired samples *t*-test) between control conditions and test conditions. The values obtained with the perifocal lenses were more negative than with the monofocal lenses, meaning that the perifocal lenses induced a myopic defocus in the spherical equivalent in the nasal retina. In the temporal retina, the mean values were less hypermetropic with the perifocal lens than the monofocal lens. However, these differences were not statistically significant. The FT also showed a statistically significant (*p*-value = 0.023, paired samples *t*-test) myopic shift in the 25N. In terms of the FS, the mean values were less hypermetropic with the test lens than with the monofocal lens, but the only statistically significant differences were in the 25N (*p*-value < 0.001, paired sample *t*-test).

**Figure 5:**
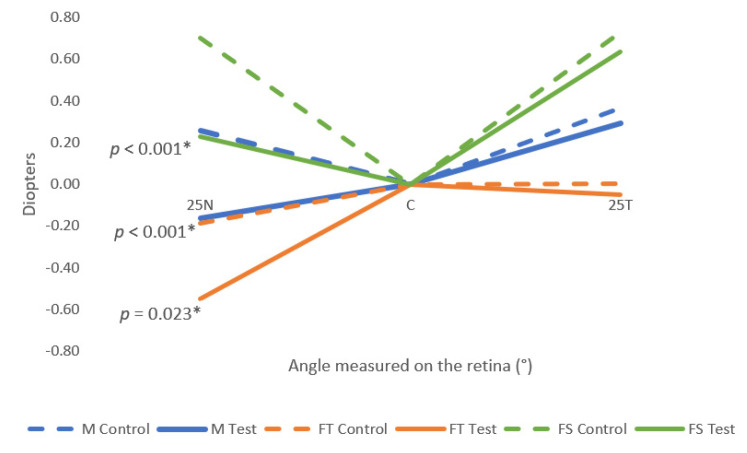
Relative mean values of M, FT, and FS for the three points measured (25T, C, 25N) for both control and test conditions. 25N: 25° eccentricity at nasal retina, 25T: 25° eccentricity at temporal retina. Standard deviation is not shown for clarity and can be consulted in Table 2. The symbol * marks the statistically significant differences (*p*-value < 0.05).

Figure 5 represents the M, FT, and FS components of the control and test conditions. All the mean values obtained in the test condition were more myopic (or less hypermetropic) than the mean values obtained in the control condition, even though only the nasal retina showed statistically significant differences.

### 3.3. Contrast Sensitivity

**Figure 6:**
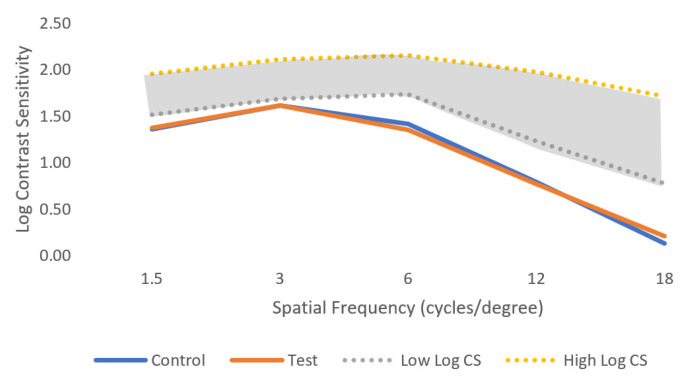
Mean of log visual contrast sensitivity for control and test conditions for the five spatial frequencies evaluated under low light conditions. The shaded area represents the normal ranges under photopic conditions for comparison. Standard deviation is not shown for clarity and can be consulted in Table 3.

Table 3 presents the results for the VCS function and for the spatial frequencies: 1.5 cpd, 3 cpd, 6 cpd, 12 cpd, and 18 cpd, with the corresponding *p*-value for each combination (control test). There were no statistically significant differences between the control and test conditions (normal ophthalmic lenses and perifocal lenses, respectively), in any of the spatial frequencies (*p*-value > 0.05, Wilcoxon test). Figure 6 represents the log contrast sensitivity in both control and test conditions in comparison with the normal values of the log contrast sensitivity (represented by the dashed lines).

### 3.4. Light Disturbance

The average values are shown in Table 4 for control and test conditions. To evaluate the light disturbance, four parameters were analyzed: LDI, BFC_Rad_, BFC_Irreg_, and BFC_IrregSD_. The perifocal lens did not shown any statistically significant changes compared with the monofocal lens (*p*-value > 0.05, Wilcoxon test and paired samples *t*-test) in the four parameters evaluated.

## 4. Discussion

### 4.1. Peripheral Refraction

In the present work, the perifocal lenses induced a significant myopic defocus in the spherical equivalent (M) in the nasal retina, at 25° eccentricity, where the peripheral refraction changed on average −0.42 D in the right eye, as shown in Table 2. In the temporal retina, influenced by the nasal side of the lenses, the values obtained with the test lens were more negative than the control lens, but the changes were not statistically significant. Considering the design of the perifocal lens, these results can be explained by the lower treatment addition power of 0.50 D in the nasal side of the perifocal lenses, which projects on the temporal side of the retina. The results of the present study also show an asymmetry between the nasal and temporal retina with the perifocal lens, as expected, as a result of this lens being specifically designed to create such asymmetry in myopic young children with progressing myopia.

Tarutta et al. (2019) described the long-term results of these perifocal defocus spectacle lenses in children between 7–14 years old with progressive myopia [13]. They studied the effect of these lenses on peripheral refraction at 15° and 30° in the nasal and temporal retina and showed a more negative peripheral refraction with perifocal lenses than the control for both nasal and temporal retina, even though in the 30° N the refraction was still positive [13]. These results are similar to the results obtained in the present study.

Recently, a study presented at the International Myopia Conference in 2022 studied the peripheral refraction of three spectacle lenses for myopia control: the DIMS, HAL, and perifocal lenses. The authors observed that the perifocal lenses induced a significant myopic shift in the relative peripheral refraction [20]. However, only the eyes of three adult participants (one emmetropic and two myopic) were analyzed, and no further information was described about the methods used for the measurements.

A study that evaluated the peripheral refraction in DIMS lenses over a two-year follow-up showed a peripheral myopic defocus induced by the DIMS, similar between the nasal and temporal retina. Contrary effects were observed in the single-vision group, which showed an asymmetrical pattern of myopic defocus shift between the nasal and temporal retina, where the nasal retina showed more hyperopic relative defocus than the temporal retina [21]. Contrary to the conclusions of that study and previous studies [14], the present results showed symmetric peripheral refraction between the nasal and temporal retina, in the naked eye condition. This might be explained by the lower sample size of the present study and the lower myopic values in terms of central refraction compared with the other studies.

The perifocal lenses used in this work include progressive addition power along the horizontal meridian beyond the central single-vision area. Berntsen et al. (2013) observed the effect of a progressive addition lens (with +2.00 D of addition) on peripheral refraction at 30° in the horizontal meridian and 20° in the vertical meridian, in 84 myopic children, in a one-year follow-up [22]. They concluded that the superior retina was more myopic with progressive addition spectacles (PAL) than with single-vision spectacles, because of the inferior near addition of +2.00 D of the PAL. They also reported that the defocus on the nasal retina was more myopic with PAL than with a single-vision lens, whereby there were no other significant changes in the other retina locations [22]. This seems logical considering that the near vision channel is located nasally to the center of the lens, causing aberrated areas in the temporal side of the lens that induce more myopic defocus in the nasal region of the retina under presbyopic PAL lenses.

In the present study, the astigmatic myopic relative peripheral refraction induced by the lens is lower than that claimed by the manufacturer and lower than previous reports. This might be explained by the fact that we evaluated the peripheral refraction at 25° in the nasal and temporal directions. At a 12 mm vertex distance from the eye (about 15.50 mm from the entrance pupil), this represents a distance of 6.90 mm to the nasal and temporal side of the lens. This is still far from the 15 to 17 mm where the maximum treatment power of the lens is incorporated. Assuming that the induced peripheral defocus is the mechanism to reduce myopia progression, and that a dose–response exists between the amount of defocus and myopia progression as recently found in contact lens studies [23], future improvements to this design might incorporate changing treatment power to determine the maximum value of induced myopization that can be obtained as a function of the pupilar diameter.

### 4.2. Contrast Sensitivity

In this study, the VCS did not suffer any changes when participants were using the perifocal lenses. Since these lenses have a central area designed to correct far distance, it was expected that the central vision would remain unchanged. As seen from Figure 6, the values obtained in both control and test conditions were below the normal values of contrast sensitivity. This happened because we tested the VCS in low light conditions to evaluate if there would be any changes under more adverse conditions than normal. It is expected that for VCS measured in photopic conditions, the values will be similar to those in the normal range.

Kaymak et al. also evaluated VCS with DIMS lenses in eight myopic subjects, both in photopic and mesopic conditions [10]. They concluded that this lens does not decrease VCS compared to a single-vision lens in both photopic and mesopic conditions. However, the peripheral contrast sensitivity was affected for both nasal and temporal meridians [10]. Similarly, Gao et al. (2021) reported no differences in peripheral VCS with HAL and SAL, compared to SVL [24]. In this study we did not measure visual contrast sensitivity when viewing through the peripheral optics of the lens.

A crossover study published in 2021 by Li et al. evaluated the short-term visual performance of 36 healthy myopic children with three lenses for myopia control: HAL, SAL, and DIMS lenses [12]. They found that HAL and SAL induced a smaller impact on contrast sensitivity than spherical lenslets displayed in a honeycomb configuration, especially for high spatial frequencies [12]. VCS was reduced in mesopic light conditions, especially in high frequencies, with the three types of lenses and this is in agreement with the results of the present study measured under low light (mesopic) conditions.

García-Marqués et al. (2020) also reported mesopic contrast sensitivity for dual-focus contact lenses [25]. They measured the contrast sensitivity between 0.01 cd/m^2^ to 3 cd/m^2^ with the VCTS 6500 in 28 healthy myopic adults. They reported a statistically significant decrease in mesopic contrast sensitivity with dual-focus contact lenses compared with single-vision contact lenses, except for the highest spatial frequency, at 18 cycles per degree [25]. This decrease can happen because of the design of this dual-focus contact lens; however, it is not expected with a perifocal lens.

### 4.3. Light Disturbance

The light disturbance analysis showed that there were no significant changes in light disturbance for the participants when they were using the perifocal lenses. This might be possible since the central area is possibly large enough that the participants did not notice more subjective glare when using the perifocal lenses compared to the monofocal lenses.

García-Marqués et al. (2020) analysed light disturbance with a dual-focus contact lens on 28 healthy myopic adults between 18 and 32 years using a light distortion analyser (LDA) [25]. They concluded that LDI, BFC_Radius_, BFC_Irregularity_, and BFC_IrregularitySD_ were higher for the dual-focus lenses when compared with a single-vision contact lens of the same material [25]. Another study analysing the perception of light disturbance in children using the same dual-focus contact lenses and the LDA device reported similar results, but this effect decreased over the two-year follow-up. For the four parameters tested, the results were better for binocular measures, similar to the findings reported in the present work [26]. These differences are expected to occur with a dual-focus contact lens because of the design of this lens with a diameter of 3.36 mm in the central distance zone, but are not expected in a perifocal ophthalmic lens such as the one tested in this study, which has a larger central distance refraction area.

In the present study, all measurements, both in control and test conditions, were obtained with three lenses placed on the trial frame, which can induce dispersion of the light and other phenomena. Using three lenses in the trial frame does not allow us to test the lenses under normal wearing conditions. However, any potential bias would be present in the different conditions so that the differential effect between them would be the contribution of the myopia control lens. The fact that the current lens design does not induce changes in LD allows us to hypothesize that subjective complaints of glare and haloes might not be reported by participants when using perifocal ophthalmic lenses. This also suggests that there is room for future optimization of the lens design, eventually reducing the central clear vision zone and/or increasing the peripheral addition to further induce higher levels of peripheral astigmatic defocus, particularly in the nasal side of the lens compared to the current design, but this needs more investigation.

## 5. Conclusions

This work showed that the perifocal lenses induced a mild level of myopic defocus in the peripheral refraction, mainly in the nasal retina (induced by the temporal side of the lens). This asymmetry is intended by the lens design. It also showed that the perifocal lenses did not lead to any reduction in the visual contrast sensitivity evaluated in low-light vision, as well as in the perception of light disturbances.

Regarding future work, it will be relevant to evaluate visual acuity both in low and high contrast conditions, as well as peripheral visual contrast sensitivity with a perifocal lens, and peripheral refraction in more retinal meridians to better understand the power profile obtained in the retina with these lenses. It also would be interesting to test variations of the present design to evaluate the effect of changing the size of the central clear zone and increasing amounts of peripheral treatment power in visual function and peripheral defocus.

## Figures and Tables

**Table 1:** Characteristics of the participants in terms of refractive error, visual acuity, and axial length. RE: right eye; LE: left eye; M: spherical equivalent; J0: astigmatic component in the horizontal meridian; J45: oblique astigmatic component; BCVA: best corrected visual acuity; AL: axial length.

	RE	LE	Binocular
M (D)	−2.80 ± 1.75	−2.81 ± 1.82	
J0 (D)	−0.03 ± 0.33	0.04 ± 0.33	
J45 (D)	0.01 ± 0.17	0.05 ± 0.20	
BCVA (LogMar)	−0.03 ± 0.06	−0.03 ± 0.08	−0.12 ± 0.06
AL (mm)	24.61 ± 0.78	24.62 ± 0.89	

**Table 2:** Mean ± standard deviation of the relative values of M, FT, and FS for the three points measured (25N, C and 25T), for the naked eye condition, and the control and test conditions. The *p*-value refers to the differences between control and test conditions.

	M				FT				FS			
	Naked Eye	Control	Test	*p*-Value	Naked Eye	Control	Test	*p*-Value	Naked Eye	Control	Test	*p*-Value
25N	−0.14 ± 0.98	0.26 ± 0.97	−0.16 ± 1.01	<0.001 *	−0.63 ± 1.23	−0.19 ± 1.11	−0.55 ± 1.19	0.023 *	0.35 ± 0.85	0.70 ± 0.92	0.23 ± 0.94	<0.001 *
C	0.00 ± 0.00	0.00 ± 0.00	0.00 ± 0.00	1.000 ^+^	0.00 ± 0.00	0.00 ± 0.00	0.00 ± 0.00	1.000 ^+^	0.00 ± 0.00	0.00 ± 0.00	0.00 ± 0.00	1.000 ^+^
25T	−0.15 ± 0.78	0.37 ± 0.87	0.29 ± 0.83	0.366 *	−0.75 ± 0.94	0.00 ± 0.96	−0.05 ± 0.93	0.660 *	0.46 ± 0.74	0.73 ± 0.83	0.63 ± 0.81	0.361 *

* paired samples *t*-test; ^+^ Wilcoxon test.

**Table 3:** Mean ± standard deviation of log visual contrast sensitivity for five spatial frequencies, for both control and test conditions. All *p*-values were calculated using the Wilcoxon test.

Spatial Frequency (cpd)	Control	Test	*p*-Value
1.5	1.36 ± 0.36	1.38 ± 0.38	0.194
3	1.61 ± 0.42	1.62 ± 0.43	0.322
6	1.42 ± 0.40	1.35 ± 0.39	0.192
12	0.79 ± 0.44	0.77 ± 0.52	0.844
18	0.13 ± 0.26	0.21 ± 0.31	0.257

**Table 4:** Mean ± standard deviation of the four parameters measured for light disturbance analysis, for both control and test conditions. The symbol * marks the *p*-value obtained with the paired samples *t*-test, contrary to the others obtained with the Wilcoxon test.

LDA Parameters	Control	Test	*p*-Value
LDI (%)	11.60 ± 6.42	10.88 ± 6.10	0.477 ^+^
BFC_Rad_ (mm)	26.85 ± 7.37	26.04 ± 7.02	0.423 *
BFC_Irreg_ (mm)	0.53 ± 0.48	0.69 ± 0.57	0.246 ^+^
BFC_IrregSD_ (mm)	4.00 ± 1.01	4.07 ± 1.69	0.868 ^+^

* paired samples *t*-test; ^+^ Wilcoxon test.

## Data Availability

The data presented in this study are available on request from the corresponding author. The data are not publicly available due to privacy reasons.
